# A Numerical Investigation of the Electric and Thermal Cell Kill Distributions in Electroporation-Based Therapies in Tissue

**DOI:** 10.1371/journal.pone.0103083

**Published:** 2014-08-12

**Authors:** Paulo A. Garcia, Rafael V. Davalos, Damijan Miklavcic

**Affiliations:** 1 Bioelectromechanical Systems Laboratory, Virginia Tech – Wake Forest University, Blacksburg, Virginia, United States of America; 2 University of Ljubljana, Faculty of Electrical Engineering, Ljubljana, Slovenia; University of California at Berkeley, United States of America

## Abstract

Electroporation-based therapies are powerful biotechnological tools for enhancing the delivery of exogeneous agents or killing tissue with pulsed electric fields (PEFs). Electrochemotherapy (ECT) and gene therapy based on gene electrotransfer (EGT) both use reversible electroporation to deliver chemotherapeutics or plasmid DNA into cells, respectively. In both ECT and EGT, the goal is to permeabilize the cell membrane while maintaining high cell viability in order to facilitate drug or gene transport into the cell cytoplasm and induce a therapeutic response. Irreversible electroporation (IRE) results in cell kill due to exposure to PEFs without drugs and is under clinical evaluation for treating otherwise unresectable tumors. These PEF therapies rely mainly on the electric field distributions and do not require changes in tissue temperature for their effectiveness. However, in immediate vicinity of the electrodes the treatment may results in cell kill due to thermal damage because of the inhomogeneous electric field distribution and high current density during the electroporation-based therapies. Therefore, the main objective of this numerical study is to evaluate the influence of pulse number and electrical conductivity in the predicted cell kill zone due to irreversible electroporation and thermal damage. Specifically, we simulated a typical IRE protocol that employs ninety 100-µs PEFs. Our results confirm that it is possible to achieve predominant cell kill due to electroporation if the PEF parameters are chosen carefully. However, if either the pulse number and/or the tissue conductivity are too high, there is also potential to achieve cell kill due to thermal damage in the immediate vicinity of the electrodes. Therefore, it is critical for physicians to be mindful of placement of electrodes with respect to critical tissue structures and treatment parameters in order to maintain the non-thermal benefits of electroporation and prevent unnecessary damage to surrounding healthy tissue, critical vascular structures, and/or adjacent organs.

## Introduction

Electroporation is a phenomenon resulting from an increase in the transmembrane potential (TMP) of a cell above a critical value leading to pore formation [1]–[3]. This threshold can be obtained by the application of an external pulsed electric field (PEF) of sufficient strength and duration [4]. Irreversible electroporation (IRE) occurs if the cell cannot recover from the membrane disruption, and eventually dies [5]–[10]. Prior to IRE, biotechnological and therapeutic applications of electroporation in tissue have avoided the irreversible regime in order to maintain high cell viability. This process of reversible electroporation has been used to successfully treat cancer when the PEFs are combined with chemotherapeutic agents or plasmid DNA [11]–[16]. The PEFs themselves are administered so as to not kill cells directly but instead aid in the uptake of molecules with lethal or therapeutic potential – this combined approach being named electrochemotherapy (ECT) or gene therapy based on gene electrotransfer (EGT), respectively [16], [17]. Recently, IRE alone without adjuvant molecules has also proven to be a safe and effective minimally invasive ablation modality with the potential to treat many currently unresectable and/or untreatable tumors [18]–[28] due to its ability to non-thermally kill substantial volumes of tissue, including tumors. The non-thermal mode of cell death (unlike in microwave or radiofrequency ablation) in IRE is unique in that it does not rely on thermal damage from Joule heating to kill tumor cells. Thus, it allows for successful treatment even in close proximity to critical structures and without being affected by the heat sink effect due to the presence of large vessels [8], [26], [29], [30].

Electroporation-based therapies involve placing electrodes within and/or around the target tissue and delivering a series of eight to one hundred short duration (∼100 µs), high voltage (∼1000–3000 V) electric pulses. Because the mechanism of cell kill in the electroporation-based therapies does not depend on thermal processes, important extracellular matrix components are spared [29], [31]–[33], including nerve and blood vessel architecture. This limits post-procedural complications and promotes a rapid re-growth of healthy tissue [32]–[34]. To verify that a specific protocol generates minimal thermal damage due to Joule heating and capitalizes on the benefits associated with a non-thermal ablation, the temperature in the tissue has been measured and calculated based on predictions of the electric field distribution [30], [35]–[40].

Accurate evaluation of the electrical and thermal responses in tissue is important to maximize the benefits from an IRE procedure. The goal in IRE is to achieve complete coverage of the targeted tissue with sufficiently high electric field while ensuring that the temperature increase during a procedure does not generate thermal damage. Although minimal thermal damage is oftentimes expected and was reported to occur near the sharp transition around the electrodes [34], recently protocols outside the traditional ∼100 pulses are being evaluated for IRE. These studies have experimentally demonstrated that if the protocols are not selected appropriately they may result in cell death due to thermal damage in addition to irreversible electroporation [41], [42]. Additionally, Scheffer *et al.* suggests that the placement of electrodes should be greater than 2 mm from the central bile ducts, pancreatic ducts, or intestines as a safety precaution [28]. Therefore, numerical evaluation of PEF protocols outside the traditional parameters and electrode configuration is important in order to provide insight as to the potential limits at which cell kill due to thermal damage could be initiated.

The aim of the current study is to characterize the electrical and thermal injury that occurs within zones of irreversible electroporation with a commercially available bipolar probe. We accomplished this by coupling the Continuity, Heat Conduction, and Arrhenius equations with a published statistical model of cell death due to PEF exposure [30], [43], [44]. Specifically, we modeled a typical IRE procedure in which ninety 100-µs pulses were delivered at a pulse repetition frequency of 1 Hz. We also varied the electrical conductivity of the liver tissue in order to capture the potential organ-to-organ variability that may be present. Our results confirm that it is possible to achieve significant cell kill due to irreversible electroporation with appropriately selected pulse parameters. Additionally, although the cell kill is primarily due to electroporation, if the pulse number and/or electrical conductivity of the tissue is too high there is also potential to achieve cell kill due to thermal damage in the immediate vicinity of the electrodes. Therefore, it is critical for the physicians and researchers to be mindful of the placement of the electrodes/probes with respect to critical tissue structures and to select pulse protocols carefully in order to maximize the benefits of this new non-thermal mode of cell kill and prevent unnecessary damage to surrounding healthy tissue, critical vascular structures, and/or adjacent organs.

## Materials and Methods

**Figure 1:**
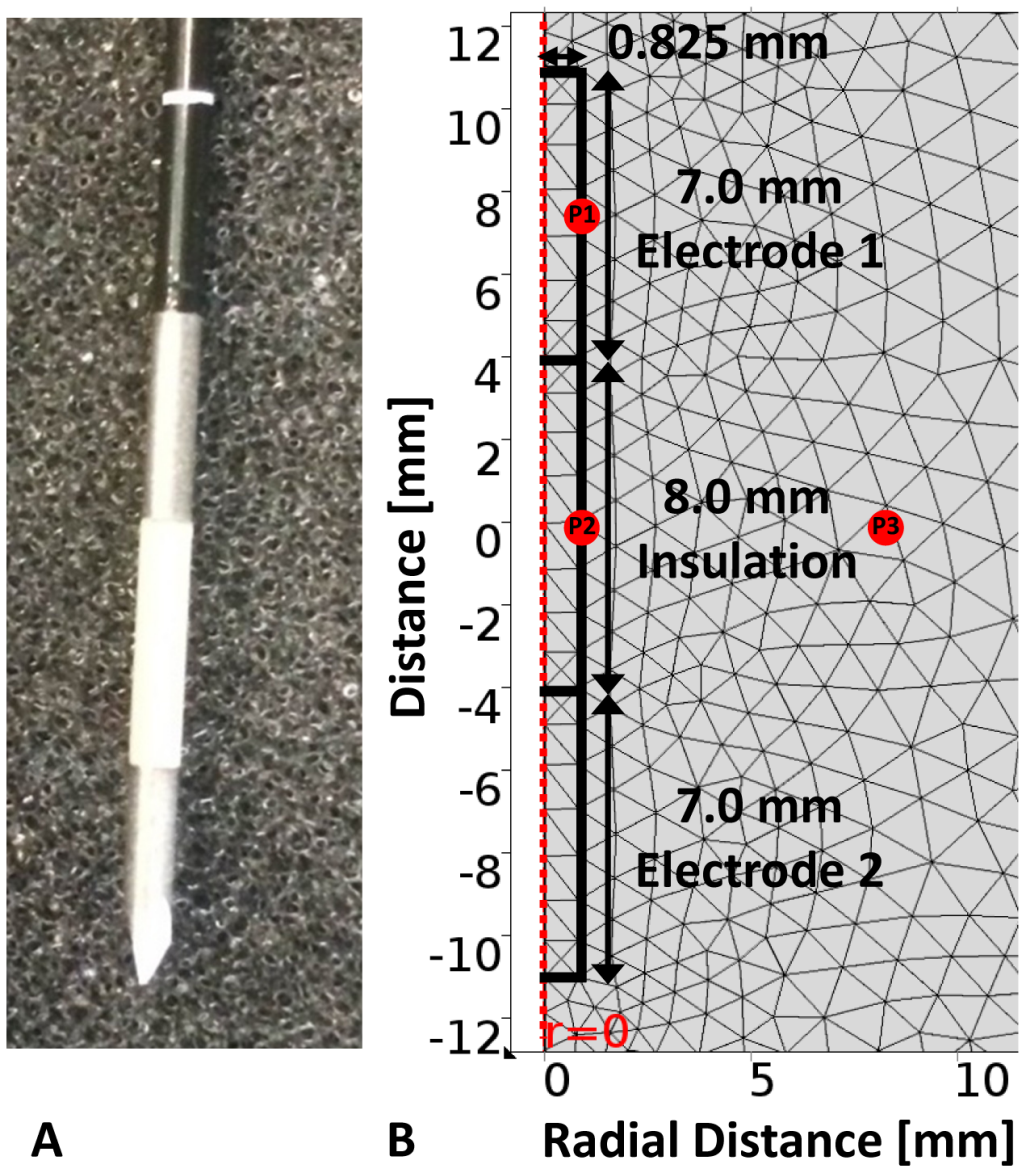
Physical electrode and mesh visualization for the extremely fine setting used in the numerical modeling software. Photograph of the physical bipolar electrode (left) and the electrode domain (right) geometry with the corresponding mesh employed in the computational modeling of the bipolar probe used in irreversible electroporation of liver tissue. The points P1, P2, and P3 depict the arbitrary locations at which the temperature was evaluated to determine the length required for thermal equilibration post-treatment.

In these simulations we modeled a commercially available electrode for irreversible electroporation of liver tissue (**Figure 1A**). Specifically, the electrodes have a bipolar configuration in which two cylindrical electrodes are fixed in one probe. The 16 gauge (1.65 mm in diameter) bipolar probe (AngioDynamics, Queensbury, NY) [45] modeled contained two 7.0-mm electrodes separated by an 8.0-mm insulation and is representative of the one used *in vivo* by Lee *et al.* during some of the initial irreversible electroporation studies [46].

### Description of the mesh and electrode configuration

The model was constructed using COMSOL Multiphysics 4.4 (Burlington, MA) with a triangular mesh that contained 14,053 elements (**Figure 1B**). The model was re-meshed until there was less than 2% change in electric field with further refinements, resulting in the extremely fine mesh setting in the numerical modeling software. The geometry of the electrode and tissue was constructed in a 2-D axis symmetric model in order to capitalize in the rotational symmetry and generate the solutions in a more computationally efficient manner than the equivalent 3-D model. The liver tissue domain was selected to be 24 cm in length (z-direction) and 12 cm in width (r-direction) which represents a 3-D cylindrical domain which is twice the dimensions of the one implemented by Chang *et al.* in order to prevent edge effects [47]. Due to rotational symmetry of the geometry, only the electrode radius (0.825 mm) was modeled as depicted in **Figure 1B**. The points investigated form a triangle and are located at the electrode-tissue boundary (P1 – electrode midpoint), insulation-tissue boundary (P2 – insulation midpoint), and 7.5 mm from P2 along the centerline of the tissue and insulation (P3) as shown in **Figure 1B**. The points P1, P2, and P3 were chosen arbitrarily to evaluate the temperature over time and determine the appropriate duration of the simulation required to capture all of the thermal effects for up to 10 min after the completion of the pulse delivery. Since numerical results may be prone to errors at the boundary of two materials due to meshing, P3 was selected to be only within the liver tissue.

### Calculation of the electric field

**Figure 2:**
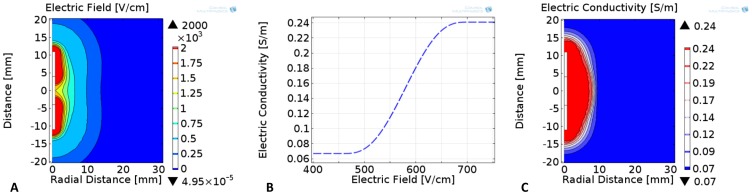
Electrical conductivity response of liver tissue during irreversible electroporation. Numerical simulation of the A) electric field and C) electric conductivity distributions during irreversible electroporation procedures with a bipolar probe and an applied voltage of 3000 V. These results employ the B) non-linear electric field dependent liver tissue properties that result immediately after the end of each electroporation pulse and was scaled by 1.25×, 1.50×, and 1.75× in order to study potential organ-to-organ variability (**Table 2**).

The electric field distribution was determined by solving the continuity equation given by:
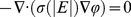
(1)where 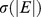 is the electric field dependent electrical conductivity of the tissue (**Figure 2B**) and 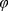 the electric potential [5], [40], [48], [49]. The electric field dependent conductivity uses a sigmoid curve with a transition zone between 460 V/cm and 700 V/cm in which the conductivity changes from 0.067 S/m to 0.241 S/m [50]. Additionally, we included cases in which the tissue conductivities were scaled by a factor of 1.25×, 1.50×, and 1.75× in order to capture the potential organ-to-organ variability. The relative error tolerance was set to 1E-5 with maximum iterations set to 100 in order to minimize any numerical instability during the non-linear electrical conductivity changes due to electroporation. In this study, the dependency of the electrical conductivity on temperature was not included since its contribution is not significant compared to changes due to electroporation alone. Nevertheless, this dependency needs to be incorporated in future studies mostly where pulse number (several hundreds) or electric fields (∼1000 V/cm) employed are high. The electrical boundary condition at one electrode-tissue interface was set to 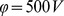 (test pulse) or 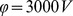 (treatment pulses) and the other electrode-tissue interface to 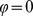. The remaining boundaries were treated as electrical insulation and mathematically described by 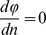.

### Calculation of the cell kill due to electroporation

The statistical model used to simulate cell kill due to electroporation was taken from a study by Golberg *et al.* [43]. This model of cell viability accounts for the number of pulses (*n*) and electric field (*E*) during electroporation-based therapies. The statistical model computes the ratio (*S*) of surviving cells (*N*) after electroporation to the number of cells prior to treatment (*N_0_*) and is given by:
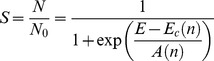
(2)where *E* is the applied electric field, 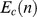 the critical electric field at which 50% of the cell population is killed, and 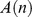 a function of the pulse number. Golberg *et al.* performed regression analysis to fit experimental data to Equation 2 that resulted in Equations 3 and 4 below:
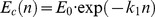
(3)

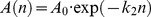
(4)where 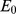 (399,600 V/m), 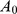 (144,100 V/m), 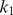 (0.03), and 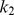 (0.06) were the regression coefficients [43]. The statistical model selected corresponded to the 100-µs pulse experimental data by Canatella *et al.* [44] in order to be consistent with the pulse duration being evaluated in this study. Since there is no available data for liver tissue, we are applying this experimental data from *in vitro* prostate cancer cells for illustrative purposes. Additionally, in order to make direct comparisons with the percentage cell kill due to thermal damage we converted the cell viability into a percentage cell kill metric given by:
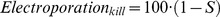
(5)


### Calculation of the temperature

The temperature distribution (*T*) within the tissue was obtained by transiently solving a modified heat conduction equation with the inclusion of the Joule heating source term,
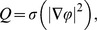


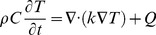
(6)where 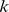 is the thermal conductivity, 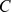 is the specific heat capacity, and 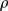 is the density of the liver tissue (**Table 1**) as reported by Chang *et al.* [47], [51]. The thermal simulation was programmed to compute the temperature during the ninety pulses by implementing a duty cycle approach that averages the temperature over the total pulse delivery time [30]. At the completion of the delivered pulse set, the simulation was programed to compute the temperature and thermal damage distributions for up to 10 minutes with a 250 ms time resolution in order to capture all the thermal effects during pulse delivery and thermal relaxation. The external tissue boundaries were all set to 37°C with the electrode-tissue and all other boundaries set to continuity.

**Table 1:** Physical properties used in the calculation of temperature and cell kill due to thermal damage in liver tissue exposed to ninety 100-µs irreversible electroporation pulses.

Physics	Symbol	Liver	Electrode	Insulation	Units	Reference
Heat	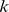	0.502	15	0.01	W/(m·K)	[28], [44], [48]
Conduction	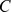	3600	500	3400	J/(kg·K)	[28], [44], [48]
Equation	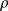	1060	7900	800	kg/m^3^	[28], [44], [48]
Arrhenius	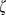	7.39×10^39^	-	-	s^−1^	[44], [52]
Equation	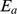	2.577×10^5^	-	-	J/mol	[44], [52]

### Calculation of the cell kill due to thermal damage

Thermal damage is a process that depends on temperature and time. If the exposure is long, damage can occur at temperatures as low as 42°C for extended exposure, while 73.4°C is considered the target temperature for instantaneous thermal damage in liver tissue as demonstrated with tissue whitening [30], [47], [52]–[54]. The damage can be calculated based on the temperatures to assess whether a particular set of pulse parameters and electrode configuration will induce cell kill due to thermal damage in superposition with cell kill due to irreversible electroporation. The thermal damage in liver tissue was quantified using the Arrhenius rate equation given by:
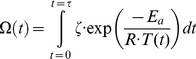
(7)where 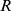 is the universal gas constant (8.314 J mol^−1^ K^−1^), 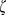 is the pre-exponential factor, a measure of the effective collision frequency between reacting molecules in bimolecular reactions, and 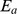 the activation energy barrier that molecules overcome to transform from their “native state” to the “damaged state” (**Table 1**) [47], [55]. It is important to note that the pre-exponential factor and activation energy are tissue specific parameters that describe different modes of thermal damage such as microvascular blood flow stasis, cell death, and protein coagulation [56]. In terms of finite element modeling of thermal damage, an integral value 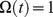 corresponds to a 63% probability of cell death and an integral value 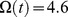 corresponds to 99% probability of cell death due to thermal effects. The computed integral is converted to a percentage value of cell kill due to thermal damage by the following equation:
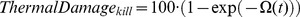
(8)The use of survival ratio for electroporation and thermal effects allows us to add both effects and get the total cell survival ratio. This relies on the assumption that mechanisms of cell death from electroporation and temperature are independent, while there might also be synergistic effects (i.e. the total survival would be smaller than predicted by this model).

## Results

The electric field distribution, one of the main factors determining the outcome of irreversible electroporation [40], [50], as well as for other electroporation-based treatments [49], [57] shows that regions surrounding the electrodes experience the highest electric fields (**Figure 2A**). The bipolar electrode configuration investigated in this study generates a non-uniform electric field that becomes weaker with increasing distance from the probe. The non-linear increase in the bulk tissue conductivity during electroporation-based therapies (**Figure 2B**) occurs when cells in tissue are exposed to high enough electric fields that compromise the cell membrane and allow for flow of ions through the cells [50], [58]. In the models, this behavior was captured numerically with the sigmoid function that was previously determined for liver tissue in rabbit using a two single electrode configuration [50] and results in an electric conductivity distribution with non-electroporated and electroporated regions (**Figure 2C**) – other functional dependencies have been investigated as well [59]. An electric field lower than 460 V/cm is insufficient to electropermeabilize the cells and the bulk tissue conductivity remains at the baseline (non-electroporated) value of 0.067 S/m. Electric field strengths greater than 700 V/cm resulted in a maximum bulk tissue conductivity of 0.241 S/m and represents the electroporated region. There is also a narrow transitional zone in between the non-electroporated and electroporated tissue which assumes values in between the minimum and maximum electric conductivities in the cases where the tissue is exposed to electric fields in between 460 V/cm–700 V/cm. These values were determined experimentally in rabbit liver tissue using eight rectangular monophasic pulses of 100-µs duration and 1 Hz pulse repetition frequency [50].

**Figure 3:**
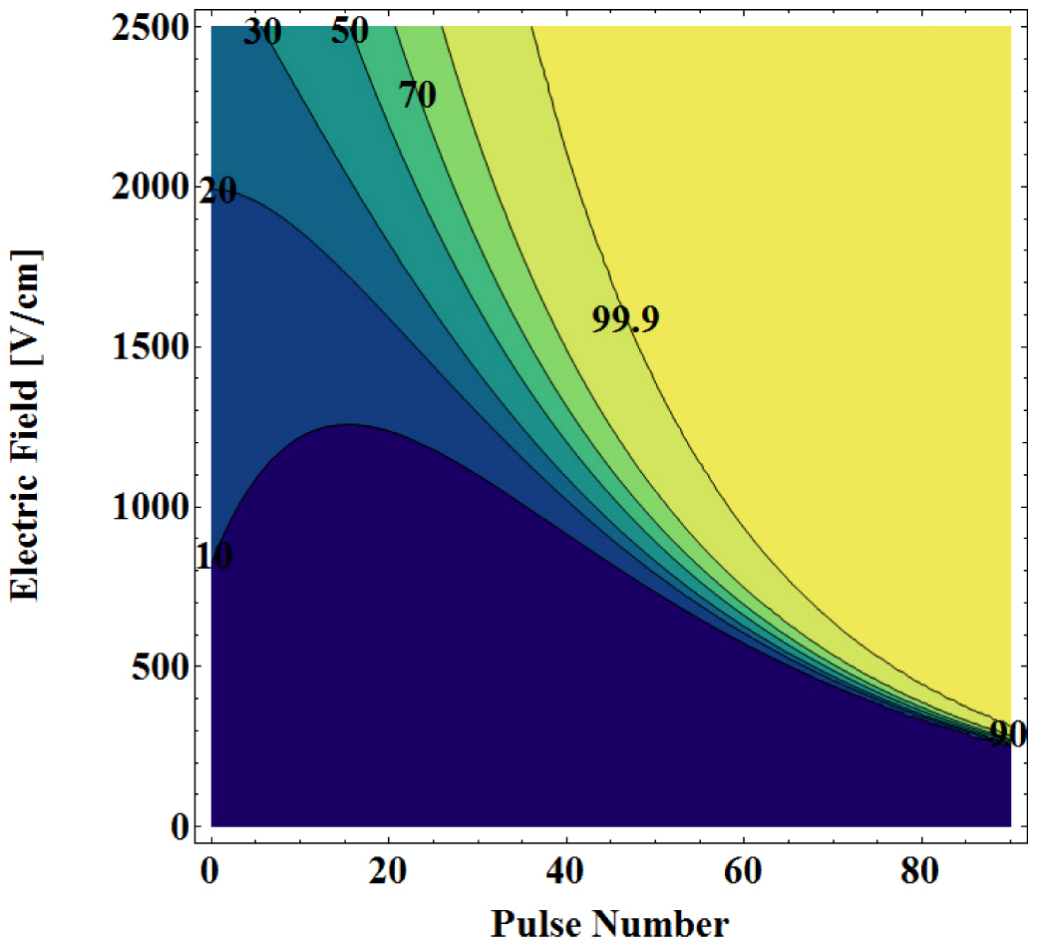
Statistical model of the probability of cell kill due to pulse number in irreversible electroporation procedures. The results demonstrate that cell kill due to irreversible electroporation is a function of electric field strength and pulse number as depicted in the 2D contour plot. Note: The data for these plots was adapted from Golberg *et al.* and demonstrate that there is a minimum electric field and pulse number needed to achieve a 99.9% probability of cell kill due to electroporation [43].

The statistical model to evaluate cell kill due to electroporation is valuable because it provides a probabilistic way of assessing the lethality of electroporation as opposed to the traditional deterministic method [60]. **Figure 3** provides the percentage of cell kill as a function of electric field strength and pulse number in a contour plot. The plot suggests that exposing the tissue to higher electric field strength and/or higher pulse number would result in a greater probability of cell kill due to electroporation.

**Figure 4:**
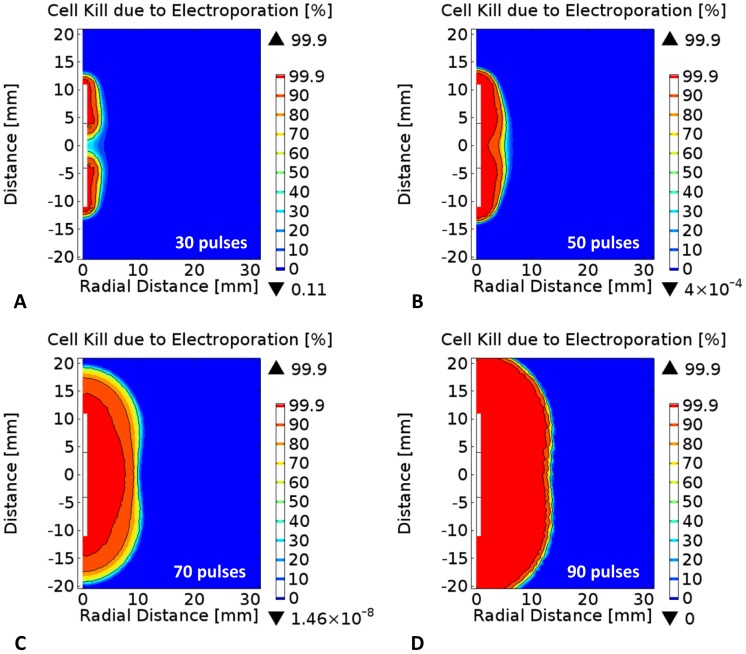
Effect of pulse number on probability of electric damage during irreversible electroporation in liver tissue. Percentage cell kill due to electroporation after A) thirty, B) fifty, C) seventy, and D) ninety 100-µs pulses using a bipolar probe with an applied voltage of 3000 V. The solid isocontours represents the 50%, 90%, and 99.9% levels from the statistical model of cell kill due to irreversible electroporation, respectively.

**Figure 4** displays the probabilistic distribution of cell kill due to electroporation as a function of pulse number. In order to visualize the increase in the probability of cell death, the 50%, 90%, and 99.9% solid black isocontour lines are provided for each of the 30, 50, 70, and 90 delivered pulses. The results demonstrate that with increasing pulse number one can achieve larger cell kill zone due to electroporation as suggested previously by Golberg *et al.* [43]. However, one thing to keep in mind is that even though more pulses will represent a higher probability of cell kill due to electroporation it will also result in increased Joule heating; thus a potentially higher probability of cell kill due to thermal damage as well. One additional aspect of these results is that with increasing pulse number, the transition zone between electroporated and non-electroporated tissue becomes sharper, replicating one of the key attributes of irreversible electroporation therapy [33], [61]. Specifically, the transition zone between the 50% and 99.9% isocontours along the centerline of the insulation changes from 2.85 mm, 2.50 mm, 2.00 mm, 1.10 mm, and 1.25 mm for 50, 60, 70, 80, and 90 delivered pulses, respectively.

**Figure 5:**
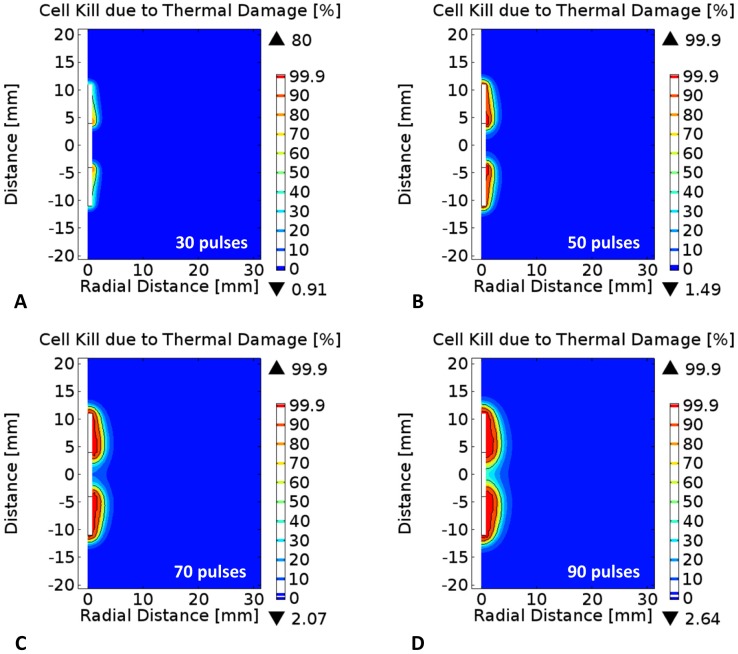
Effect of pulse number on probability of thermal damage during irreversible electroporation in liver tissue. Percentage cell kill due to thermal damage after A) thirty, B) fifty, C) seventy, and D) ninety 100-µs pulses using a bipolar probe with an applied voltage of 3000 V. The solid isocontours represents the 50%, 90%, and 99.9% levels from the statistical model of cell kill due to thermal damage, respectively.

**Figure 6:**
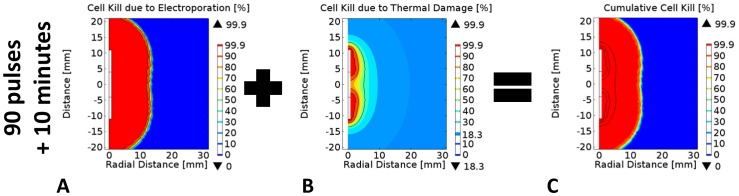
Probability of cell kill due the cumulative effects of electroporation and thermal damage. Cell kill due to A) electroporation only, B) thermal damage only, and C) combined damage effects 10 minutes after the completion of the ninety 100-µs pulses delivered at a pulse repetition frequency of 1 Hz. Note: The solid black curves correspond to the 50%, 90%, and 99.9% cell kill isocontours.

**Figure 5** and **6** provide additional results that are representative of IRE procedures in which tissue is generally exposed to at least ninety pulses. The pulse repetition frequency was set to 1 Hz due to the synchronization of the pulses with the heart rate that is used in clinical practice to prevent inducing cardiac arrhythmias [62]. In this simulation, the applied voltage was set to 3000 V and the pulse duration to 100 µs, in order to study the upper limit of the cell kill due to electroporation and thermal damage since these are the maximum programmable parameters in the NanoKnife system (AngioDynamics, Queensbury, NY) [63]. The results from the simulations suggest that starting at 30 pulses, the increase in temperature during electroporation results in the onset of cell kill due to thermal damage at the electrode-insulation interface. The cell kill due to thermal damage continue to grow as more pulses are delivered due to the cumulative exposure of the tissue to elevated temperatures. At the completion of the ninety pulses, there seems to be significant cell kill due to thermal damage surrounding the electrodes as shown in **Figure 5D**. These results are important and should be considered by clinicians/surgeons as positioning electrodes sufficiently far way from sensitive tissue components will allow the prevention of thermal damage to critical structures or organs in close proximity to the treatment zone.

**Figure 6A–6C** shows the cell kill distributions for the tissue 10 min after the completion of ninety 100-µs electroporation pulses. From the probabilistic distributions of cell kill it is evident that with these specific pulse parameters, a significant amount of tissue is killed by irreversible electroporation in a non-thermal manner (**Figure 6A**). However, a smaller but still significant region surrounding the electrode is killed by thermal damage due to exposure to elevated temperatures (**Figure 6B**). As a result, it is important for physicians and researchers to be aware of the potential modes of cell kill that can be achieved with PEF in order to prevent damage to critical structures or organs in close proximity to the treatment zone. Placement of the electrodes thus needs to be at a safe distance from these critical structures to avoid their thermal damage. Consistent with our findings, Scheffer *et al.* recently recommended placing the electrodes at a distance greater than 2 mm from central bile ducts, pancreatic ducts, and intestines to avoid deleterious thermal effects [28].

**Figure 7:**
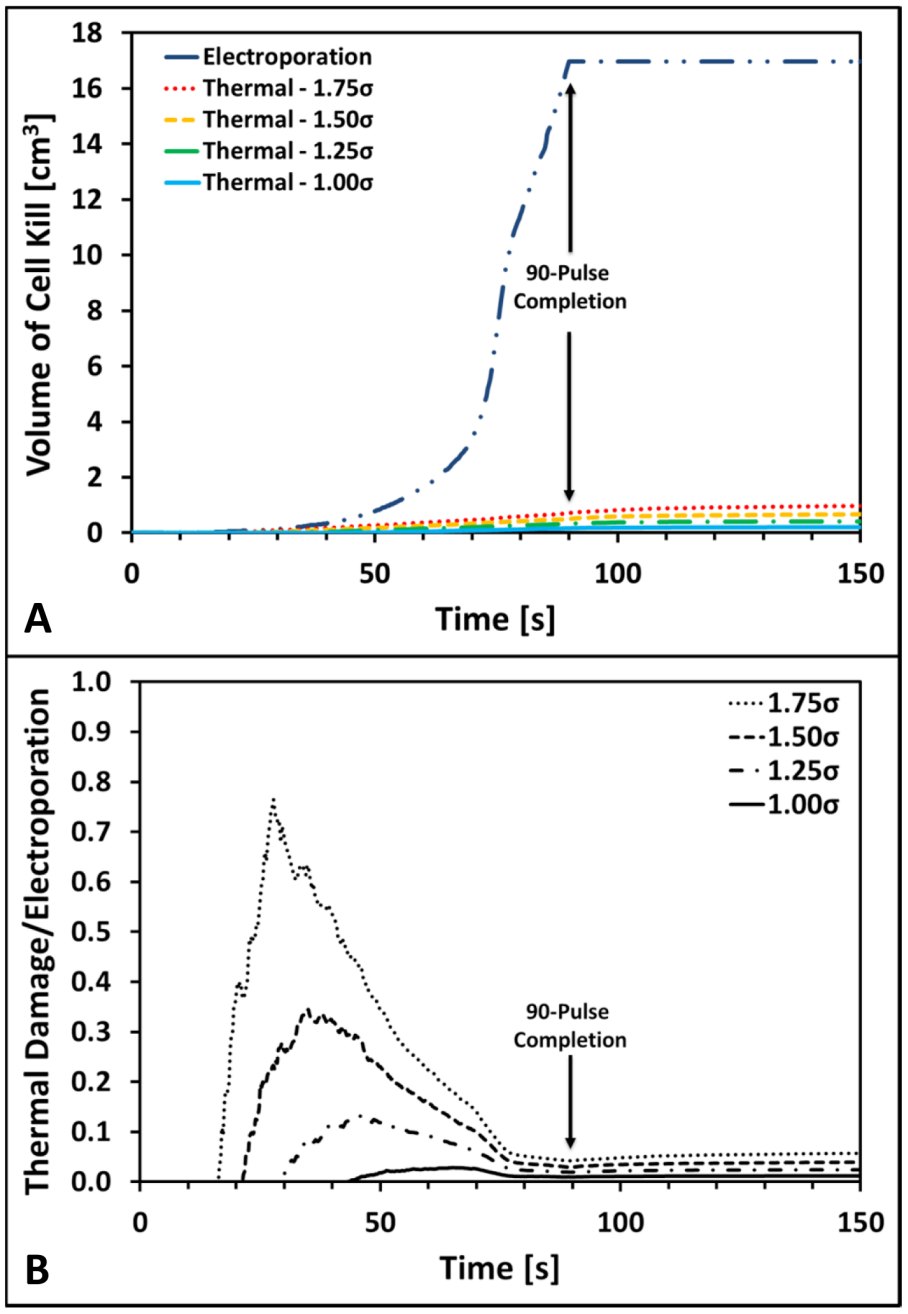
Volumes of cell kill due to irreversible electroporation and thermal damage in liver tissue. Panel A displays the computed volumes of cell kill due to electroporation and thermal damage for each of the four baseline electrical conductivities investigated. Panels B shows the curves quantifying the ratio of cell kill due to thermal damage and electroporation as a function of conductivity as well. The results in panels A and B were computed during and after the delivery of a ninety 100-µs pulse electroporation protocol with an applied voltage of 3000 V at a pulse repetition frequency of 1 Hz.

**Figure 7** provides the results quantifying the volumes of tissue killed due to irreversible electroporation and thermal damage using a 99.9% lethal level. **Figure 7A** displays the calculated volumes of tissue killed by irreversible electroporation that increases until 16.97 cm^3^ at the completion of the pulse delivery. The calculated volume of cell kill due to electroporation then remains constant since no more pulses are delivered; however, with increasing pulses the volume could potentially increase further. In terms of the volume of cell kill due to thermal damage, there is a significant dependency on the baseline conductivity as expected since higher electrical conductivities translate into more significant Joule heating. In order to help guide physicians performing these treatments, we have also calculated the expected current from the 500 V test pulse and 3000 V treatment pulses for each baseline conductivity (**Table 2**). The computed ratio suggests that for all conductivity values investigated the volume of tissue killed by thermal damage did not surpass 6.1% of the volume of tissue treated with irreversible electroporation. Therefore, these results may provide some insight as to expected electric and thermal cell kill distributions based on the current measurements of the test pulse [64] which is already implemented in the NanoKnife pulse generator.

**Table 2:** Volumes of tissue killed by thermal damage and irreversible electroporation 10 min after a ninety 100-µs pulse protocol.

Factor	σ_min_ [S/m]	σ_max_ [S/m]	*I*_500 V_ [A]	*I*_3000 V_ [A]	Thermal Damage [cm^3^]	Irreversible Electroporation [cm^3^]	Thermal/IRE
1.00	0.067	0.241	0.45	8.35	0.22	16.97	1.3%
1.25	0.084	0.302	0.56	10.43	0.44	16.97	2.6%
1.50	0.101	0.362	0.67	12.52	0.72	16.97	4.2%
1.75	0.117	0.422	0.78	14.61	1.04	16.97	6.1%

**Figure 7B** displays the computed ratio of cell kill volumes due to thermal damage normalized by the cell kill volume due to electroporation from **Figure 7A**. There are three main aspects that should be highlighted from these results. First, since the computed ratio is always less than one, the volume of cell kill due to thermal damage is always smaller than that of the irreversibly electroporated tissue. Secondly, it is clear that with higher baseline conductivities the onset of thermal damage occurs with fewer pulses and generates a higher volume of affected tissue. The onset of thermal damage started when 43 (1.0×), 29 (1.25×), 21 (1.5×), and 16 (1.75×) pulses were delivered for the 0.067, 0.084, 0.101, and 0.117 S/m baseline electrical conductivities, respectively. Finally, there appears to be a significant thermal component in the volume of tissue treated by irreversible electroporation at lower pulse number. Nevertheless, these ratios must be considered several minutes after the completion of the pulses in order to assess all the electric and thermal effects of the treatment when lesions are fully developed. Consequently, these results support the fact that irreversible electroporation therapies are predominantly non-thermal in nature but there could be some critical locations surrounding the electrodes that may result in thermal damage due to elevated temperatures. This potential effect should also be taken into account in the geometrical design of electrodes in addition to the materials implemented to mitigate thermal damage [65].

## Discussion

Irreversible electroporation (IRE) is an electroporation-based therapy that is under clinical evaluation for the treatment of internal tumors [66], [67]. This therapy uses pulsed electric fields to permeabilize cells' membranes to induce cell death of the tissue/tumor. Cell death occurs due to a loss of homeostasis soon after the cell is permeabilized or even hours after the membrane reseals due to excessive ion transport across the membrane [9], [10], [68]. Electroporation-based therapies have shown their safety and efficacy as a cancer treatment in tumor models and clinical trials [15], [69]. Nevertheless, as the technology evolves it is important to understand other effects that might be playing a role during the delivery of the pulses. The main focus of this study was to evaluate the probability of cell kill due to electroporation and thermal damage in liver tissue during irreversible electroporation. Since the tissue is being exposed to sufficiently high electric fields to electroporate the cells, the tissue temperature will also increase due to Joule heating. Therefore, in this study we specifically evaluated the cell death due to electroporation and thermal damage achieved with a commercially available bipolar electrode (AngioDynamics, Queensbury, NY). We investigated the effect of baseline electrical conductivity within a simulated protocol in order to provide insight as to the modes of cell kill that can be achieved in IRE. Specifically, we delivered a series of ninety 100-µs pulses to liver tissue at a pulse repetition frequency of 1 Hz and estimated how the organ-to-organ variability in electrical conductivity affects the IRE treatment outcome.

The numerical results are based on the statistical models of cell kill due to electroporation and cell kill due to thermal damage implemented in the simulations. As expected, increasing the baseline electrical conductivity resulted in a significant increase in temperature due to the higher Joule heating rate during each pulse. It should be noted that thermal effects via focal therapies are typically accounted for through the use of the Pennes' bioheat equation [47], which will take into consideration other physiological phenomena such as blood perfusion and metabolic heat generation [47]. The results presented here can be considered conservative since Equation 6 does not account for heat loss due to perfusion. We have elected to neglect potential perfusion effects in this study because of the presence of vascular lock during electroporation [70], [71]. The exposure of liver tissue to elevated temperature resulted in a higher probability of cell kill due to thermal damage in the immediate vicinity of the electrodes when using an IRE protocol that employed ninety 100-µs pulses. The results also demonstrate that with increasing exposure (e.g. more pulses) to sufficiently strong electric field, larger volumes of cell kill can be achieved with irreversible electroporation. Therefore, it can be concluded that the combination of pulse number with respect to baseline conductivity must be optimized in order to minimize cell kill due to thermal damage and capitalize on the many benefits of a non-thermal therapy. We propose using the test pulse approach in which the current is measured at a non-therapeutic voltage in order to determine the baseline conductivity of the tissue [64]. Based on this value, the physician would then deliver a specific pulse number that is known to generate minimal cell kill due to thermal damage, potentially let the tissue cool down, and deliver additional sets of pulses as needed. Even though pulse number is a parameter that could be optimized to minimize thermal damage, future work should also investigate pulse duration since this parameter could provide another avenue to achieve similar cell kill distributions due to electroporation with less or virtually no thermal damage.

The statistical model of cell death due to electroporation used in this manuscript was originally suggested by Golberg *et al.* [43] with published experimental data from Canatella *et al.* for prostate cancer cells *in vitro* [44]. The statistical model was extrapolated from experimental data from DU 145 prostate cancer cells exposed to electric field strengths (100 V/cm–3,300 V/cm), pulse durations (50 µs–20 ms), and pulse numbers (1 pulse–10 pulses) to values that are more relevant to IRE treatment protocols [44]. It should be noted that this is based on *in vitro* data, which typically requires higher field amplitudes to achieve an effect than cells in tissue. It is important to note however that this model was used in absence of experimental data on cell kill due to irreversible electroporation in tissue and we simulated pulse protocols outside of the published parameters described above.

Even though we believe these results are helpful in guiding physicians and researchers as to the selection of pulse protocols, our numerical results must be validated experimentally for different tissues and tumors *in vivo*. Additionally, a model that also discriminates between electroporation-based pulses delivered at different frequencies must be developed in order to incorporate the potential impact of delivering the pulses too quickly (e.g. no pore resealing), too slowly (e.g. electrosensitisation) [72]–[74], or the effect of temperature on pore resealing [75], [76]. Finally, other modes of cell kill such as electrochemical treatments due to supraphysiological pH fronts in the immediate vicinity of the electrodes are possible that were not included in this analysis but could be considered in future work as well [77], [78].

Experimentally, IRE has shown a sharp demarcation between treated and non-treated tissue for a particular cell type [33], [34], [79]. However, if there are multiple cell types present, a statistical distribution could be used to determine which types of cells are killed and which are not affected. Furthermore, due to other biological microstructures present, the field distribution will not completely follow the solution to the Continuity equation and in some cases, it could go beyond or fall within the theoretical value, adding complexity [49], [71], [80], [81]. Therefore, the statistical model of cell death is a valuable step since it allows for partially capturing this transition zone in which cells are being continuously exposed to PEF and seems to become sharper with increasing number of pulses as calculated from our results.

## Conclusions

We used statistical models of cell kill due to electroporation and thermal damage to evaluate irreversible electroporation protocols in liver tissue. In this manner, we provide insight for physicians and researchers to evaluate pulse protocol selection allowing them to achieve the desired outcome. Evidently with pulsed electric fields one can achieve successful irreversible electroporation with or without thermal damage surrounding the electrodes. The results presented are from theoretical cases of cell kill due to electroporation and thermal damage; therefore, experimental *in vivo* validation is still necessary for implementation of this modeling approach in imaging-based treatment planning models [21], [82], [83]. Nevertheless, this manuscript confirms that all pulse parameters are synergistic and can be optimized to achieve different clinically relevant outcomes in irreversible electroporation therapies.
